# Evaluating the knowledge and training of forensic pathologists and registrars performing forensic radiography at a forensic pathology mortuary in the Free State province, South Africa

**DOI:** 10.4102/hsag.v27i0.2014

**Published:** 2022-10-27

**Authors:** Je’nine Horn-Lodewyk, Belinda van der Merwe, Gift M. Kali, Leonie Munro

**Affiliations:** 1 Department of Clinical Sciences, Faculty of Health and Environmental Sciences, Central University of Technology, Bloemfontein, South Africa; 2 Private, Durban, South Africa

**Keywords:** forensic radiography, knowledge, training, radiation protection, quality control tests

## Abstract

**Background:**

Forensic radiography is an important component in forensic sciences. There seems to be a lack of recent studies in the literature on the knowledge and training of forensic pathologists and registrars performing forensic radiography at forensic pathology mortuaries in South Africa.

**Aim:**

To evaluate the knowledge and training of forensic pathologists (consultants) and registrars performing forensic radiography at a forensic pathology mortuary in the Free State province, South Africa.

**Setting:**

A prospective study was conducted at a conveniently selected forensic pathology mortuary located in the Free State province of South Africa.

**Method:**

Personnel performing forensic radiography at the selected mortuary were invited to participate in the study. An exploratory quantitative study design was used. The research tool was a self-administered questionnaire comprising open- and closed-ended questions. Four registrars and four consultants (*n* = 8) completed the questionnaire.

**Results:**

Training was only received on computed tomography (*n* = 1; 12.5%), the C-arm machine (*n* = 1; 12.5%) and the digital X-ray mobile machine (*n* = 1; 12.5%) Lodox on corpse positioning (*n* = 7; 87.5%) and setting of exposure factors (*n* = 2; 25%).

**Conclusion:**

Lack of training of the personnel performing forensic radiography, at the selected mortuary was identified. Training is required in image acquisition protocols, quality control tests of the X-ray machines, setting technical factors and operation of various X-ray machines.

**Contribution:**

Training of registered radiation workers who perform forensic radiography in mortuaries is essential to produce high-quality ionising radiation images and ensure their own and other staff members’ safety.

## Introduction

In recent years, forensic radiography has seen significant advances and evolved into a powerful tool in forensic medicine (Obafunwa et al. 2015; Schuliar & Knudsen 2012). Forensic pathologists use forensic radiography to provide information on a deceased person’s autopsy findings and to solve criminal cases. Forensic radiography examinations are performed on the deceased (i.e. a corpse) and living persons, for example, in cases of child, spouse or elderly abuse (Kahana & Hiss 1999; Rohringer et al. 2020). For the purpose of this article, forensic radiography refers to radiographic examinations of the deceased. In South Africa, it is the promulgated responsibility of the health department in each province to provide forensic services, and among others, to conduct a diagnostic internal postmortem examination (South African Government 2004).

All medicolegal postmortem examinations are performed by a forensic medical practitioner (FMP), who could be a qualified forensic pathologist, registrar (resident, or forensic pathologist-in-training) or a medical officer, to establish the cause of and circumstances surrounding death (Dempers et al. 2018). Viner (2005) concluded that forensic radiography in South Africa was mainly performed by pathologists and, in some instances, police technicians. Importantly, in most cases diagnostic radiographers who were undertaking forensic radiological examinations lacked the training demanded for that specific type of work. Furthermore, at the Salt River mortuary in Cape Town, prior to obtaining a mobile C-arm fluoroscopy unit, forensic cases that required forensic radiography were transported to Groote Schuur Hospital for imaging on the newly acquired digital imaging Lodox Statscan. To provide a forensic radiography imaging service in the forensic pathology mortuary at Salt River would require the daily presence of a radiographer. The lack of resources (funds) and radiographers has resulted in pathologists performing fluoroscopy themselves, although they had received insufficient training on X-ray imaging equipment (Viner 2005).

Forensic radiography assists in judicial investigations as a subfield of forensic science and medicine (Elifritz et al. 2014). The courts in South Africa do not define standards of expertise for imaging by radiographers or forensic pathologists (K. Bolton [Associate Professor of Paediatrics, University of Witwatersrand, Johannesburg] pers. comm., 09 May 2022). According to Bolton, within the adversarial nature of the South African system, if a forensic pathologist gives evidence in a criminal case (e.g. for the State) on an X-ray that he or she has taken or interpreted, the defence counsel will interrogate the experience and expertise of the pathologist in this area. The defence counsel may use their own expert to give another opinion. The technical quality of the study will often be crucial. Therefore, it is important for pathologists performing forensic radiography to be trained to produce acceptable images. It is crucial that whoever performs the procedure must be trained in protecting themselves and their staff members from excess radiation (K. Bolton, pers. comm., 09 May 2022).

Consequently, it is important to highlight that personnel performing forensic radiography should have thorough training in the use of the imaging equipment and how radiographs must be taken. Radiographers who are familiar with and trained in forensic radiography might not always be available (Wilson, Bonner & Rutty 2004). Possible reasons for the unavailability of radiographers could be that they are not being trained in forensic radiography, posts are not available and radiographers prefer not to perform forensic radiography because of the physical and emotional challenges (Sangonuga, Kekana & Eze 2022).

Photography, radiography and light microscopy are standard imaging methods used in forensic investigations (Aalders et al. 2017). Technological advances have allowed for the development of new, more powerful imaging methods for forensic purposes, such as computed tomography (CT) and magnetic resonance imaging (MRI), and also expanded the scope of imaging in forensic radiography (Sangonuga et al. 2022). Aspects of acquiring imaging that influence image quality may also be considered, including the acquisition protocol and the personnel who perform forensic radiography, such as radiographers or forensic technologists who receive in-service training.

Radiographers who perform forensic radiography, for example, are required to complete postgraduate training and should be registered with the International Association of Forensic Radiographers and the Society of Radiographers in Australia and the United Kingdom (Johnson 2014). These professional organisations offer postgraduate training and seminars for forensic radiographers to develop the competence necessary to produce high-quality diagnostic images (Sangonuga 2020). Forensic radiographers should adhere to protocols in order to produce high-quality images that will be accepted in a criminal court (Kudlas, Odle & Kisner 2010; Johnson 2014).

Personnel performing forensic radiography have been identified as radiographers trained in the specific imaging modality or autopsy technicians certified in-service (Aalders et al. 2017). However, specific protocols applicable to forensic radiography have yet to be standardised (Karalis & Denton 2016). There is limited information available in the literature within the South African context on the specific training that forensic radiographers or personnel performing forensic radiography have to undergo, the knowledge required regarding the equipment used and the maintenance of forensic radiology equipment. The standard or level of knowledge needs to be clearly defined.

The Colleges of Medicine of South Africa (CMSA) is the national examining body for the medical professions of 29 constituent colleges, including the College of Forensic Pathologists of South Africa. The latter offers one specialist or fellowship and three diploma qualifications, including (1) Fellowship of the College of Forensic Pathologists of South Africa – FC for Path(SA); (2) Diploma in Forensic Medicine of the College of Forensic Pathologists of South Africa – Clin: Dip For Med(SA) Clin; (3) Diploma in Forensic Medicine of the College of Forensic Pathologists of South Africa – Clin/Path: Dip For Med(SA) Clin Path; and (4) Diploma in Forensic Medicine of the College of Forensic Pathologists of South Africa – Path: Dip For Med(SA) Path (T Naidoo [President of the College of Forensic Pathologists of South Africa] pers. comm., 17 May 2022).

Forensic imaging is embedded in the general overall learning outcomes, detailed learning objectives and respective logbook requirements of the aforementioned examinations. However, the related technical instruction is provided by the individual training institutions across the country (i.e. Health Professions Council of South Africa accredited University Departments of Forensic Medicine or other Clinical Forensic Medicine Departments, where applicable). The practical training in operational and diagnostic forensic radiology is therefore variable and not standardised (T. Naidoo, pers. comm., 17 May 2022).

The key user training is provided by Lodox to forensic pathologists at the site. In some cases, Lodox equipment training may be extended to the forensics officers (W. Papane [Sales and Marketing Executive, Lodox] pers. comm., 09 May 2022). At the forensic mortuaries where Lodox equipment has been installed (listed in Table 1), no radiographers are employed. With the Lodox, it is not necessary to manually select the exposure factors. However, the procedure (i.e. full-body antero-posterior [AP]) and the size (i.e. weight) selection are the two factors that will determine the kV and mA used. The exposures are preset according to the weight and procedure protocol selected. Changing the kilovolt peak (kVp) and milliampere-second (mAs) is not required (B. Nkosi [Clinical Applications Specialist Lodox] pers. comm., 17 May 2022).

**TABLE 1:** List of South African forensic mortuaries that use Lodox equipment and date of installation.

Province	Date of installation
**Western Cape**	
George Forensics	11 May 2015
Tygerberg Mortuary	12 April 2020
Salt River Forensics	31 March 2015
Observatory Forensic Pathology Institute	03 May 2022
**Gauteng**	
Germiston Forensics	12 December 2011
Pretoria Forensics	06 June 2014
Hillbrow Forensics	25 August 2014
Ga-Rankuwa Forensics	25 February 2015
**KwaZulu-Natal**	
Pinetown Medico Legal Mortuary	12 April 2021
Richards Bay Forensics	26 July 2016
Phoenix Forensics	28 July 2016
**Free State**	
Bloemfontein Forensic Pathology Service (FPS)	22 January 2020
**Limpopo**	
Polokwane FPS	12 March 2020
**Mpumalanga**	
Middelburg FPS	02 December 2016
Ermelo FPS	11 November 2016
Themba FPS	12 March 2020

*Source*: Personal communication, 09 May 2022; Mr W. Papane, Sales and Marketing Executive Lodox. The North West province is currently under administration, and funding to install Lodox equipment is a challenge. In the Northern Cape province, no mortuary has been identified for the installation of a Lodox system. The Eastern Cape province has just received delivery of two systems for Brighton and Mdantsane forensic facilities. Lodox is currently awaiting site renovations and the Directorate: Radiation Control ‘may install’ licences before proceeding with the installation. Future planning includes Lodox installations also at Gqeberha (Port Elizabeth) and Komani (Queenstown). KwaZulu-Natal is also awaiting one system for the Park Rynie Mortuary in 2022.

The guidelines of the Directorate: Radiation Control (DRC) indicate that in South Africa, only radiographers and radiologists registered with the HPCSA and chiropractors registered with the Allied HPCSA (AHPCSA) may operate X-ray equipment for medical use in their scope of practice (Department of Health 2016). However, in the code of practice for users of nonmedical equipment, the operators may be persons either qualified in radiography and radiology or ‘appropriately trained forensic personnel’ (Department of Health 2014). The licence holder of the forensic radiology equipment must ensure that it is used and maintained by competent, accountable and professionally skilled personnel. The licence holder must ascertain that radiation quality control (QC) tests are conducted at prescribed frequencies to monitor the forensic radiology imaging equipment’s safe and correct use (Department of Health 2014, 2016). The licence holder must also ensure that radiation levels are monitored and within appropriate levels in the work environment.

The Western Cape Forensic Pathology Service currently has 18 forensic mortuaries that vary in size and function, from the two large academic centres in Cape Town to small rural facilities (G. Kirk [Clinical Unit Head – Forensic Pathology Service (FPS), Western Cape Government, and Senior Lecturer, Division of Forensic Medicine and Toxicology, Department of Pathology, Faculty of Health Sciences, University of Cape Town] pers. comm., 22 April 2022). The larger facilities such as those in Cape Town, George, Paarl, Knysna and Worcester have their own radiology equipment, while other facilities use the local provincial hospital radiology departments. In the Western Cape FPS, all radiological examinations must be carried out by a medical practitioner, although they can be assisted by a forensic pathology officer (G. Kirk, pers. comm., 22 April 2022).

The Gauteng northern region has three forensic pathology facilities (S. Rossouw [Acting Head, Department of Forensic Medicine, University of Pretoria] pers. comm., 06 May 2022). The Pretoria facility is affiliated with the University of Pretoria, the Ga-Rankuwa facility is affiliated with the Sefako Makgatho Health Sciences University and a small mortuary in Bronkhorstspruit is staffed by a sessional medical officer (S. Rossouw, pers. comm., 06 May 2022).

There are 11 forensic mortuaries in the Free State province (Table 2). Forensic imaging equipment is only available at the mortuary in Bloemfontein. Therefore, corpses from the mortuaries in Welkom, Kroonstad and Sasolburg are transferred to the Bloemfontein forensic mortuary for forensic radiography examinations (O. Kopane [Mortuary Manager, Welkom, Kroonstad and Sasolburg] pers. comm., 27 April 2022). The researchers were not able to obtain information pertaining to the knowledge and training of personnel at these mortuaries and deemed it important to address this gap.

**TABLE 2:** List of forensic mortuaries in the Free State province with regions.

Facility	Region
Bloemfontein	Free State, Southern
Botshabelo Holding Facility	Free State, Southern
Jagersfontein Holding Facility	Free State, Southern
Smithfield Holding Facility	Free State, Southern
Bethlehem	Free State, Eastern
Phuthaditjhaba	Free State, Eastern
Harrismith Holding Facility	Free State, Eastern
Ficksburg Holding Facility	Free State, Eastern
Welkom	Free State, Northern
Kroonstad	Free State, Northern
Sasolburg	Free State, Northern

*Source*: Personal communication, 22 April 2022; Mr N. Motau-Modise, personal assistant to the Head of Department: Forensic Medicine, Free State Provincial Department of Health.

In other words, in South Africa, no nationally agreed curriculum or coordinated forensic radiography training has been proposed. The aim of this study was therefore to determine the level of knowledge and training of forensic pathologists (consultants) and registrars (forensic pathologists-in-training) who perform forensic radiography at a selected forensic pathology mortuary in the Free State province of South Africa.

## Methods

### Study design and setting

A prospective descriptive, exploratory quantitative study was conducted. Data were collected from personnel performing forensic radiography employed at a conveniently selected forensic pathology mortuary located in the Free State province of South Africa.

### Population

The target population comprised medical personnel who perform forensic radiography, excluding radiographers, employed at forensic pathology mortuaries in South Africa. However, there is not a record of the total number of this population. For the purpose of this study and because of the restrictions of the coronavirus disease 2019 (COVID-19) pandemic, the accessible population consisted of eight full-time employees involved in imaging at a conveniently selected forensic pathology mortuary in the Free State province, South Africa.

### Sample size determination and sampling technique

Purposive sampling is the purposeful selection of respondents based on their ability to provide insight into a specific subject, concept or phenomenon (Etikan 2016; Robinson 2014). Furthermore, selecting research subjects for purposeful sampling is iterative, as opposed to starting from a pre-established sampling frame (Robinson 2014). In view of the small study population, the researchers decided to invite all personnel performing forensic radiography at the selected site to participate in the study. The inclusion criteria were as follows: personnel who, at the time of the study, were performing forensic radiography or who had done so in the recent past. Inclusion criteria were not based on specific academic qualifications of the personnel, as their disciplines were unknown. Purposive nonprobability sampling was used because the population was small and specific. Literature reports the use of purposive sampling in a study that explored the experiences, attitudes and knowledge of radiographers and forensic pathologists in delivering forensic radiography services in Nigeria (Sangonuga et al. 2022). Purposive nonprobability sampling is used when limited research resources are available and because of time constraints (Etikan & Bala 2017).

### Data collection

Before commencement of the study, the participants signed an informed consent form. The data collection instrument was a purpose-designed questionnaire with open- and closed-ended questions. Relevant information pertaining to forensic radiography in the *Code of Practice for Users of Medical X-Ray Equipment* (Department of Health 2016) and *Requirements for Licence Holders with Respect to Quality Control Tests for Diagnostic X-Ray Imaging Systems* (Department of Health 2015) was adapted for use in the questionnaire (research instrument). The research instrument comprised three parts. Part 1 consisted of seven closed-ended questions to obtain demographic data. Part 2 comprised 28 both open- and closed-ended questions. Part 3 was an open-ended question for the participants to provide any other feedback when completing the questionnaire.

A copy of the questionnaire was placed in sealed envelopes and hand-delivered to the eight participants on 13 August 2020. The researchers requested them to not discuss or share their answers to the questions with other participants. This was done to reduce the risk of those who had completed the questionnaire influencing other participants’ responses when they completed the questionnaire. The completed questionnaires were collected on 04 September 2020.

### Pilot testing of the data collection instrument

Reliability of a research method refers to the probability of obtaining similar results if a study were repeated by other researchers (Bush 2012; Hartas 2010). Because the participants in this study were not asked to express opinions, it means that similar quantitative data should be obtained if the research instrument were used by other researchers. No reliability coefficient was calculated.

The content validity of the questionnaire determines whether an instrument is measuring what it is intended to measure (Delport & Roestenburg 2011). The regulatory guidelines were used as a benchmark in the research instrument to measure training and knowledge related to forensic radiographic imaging. A forensic radiographer with over 10 years’ experience and a lecturer from an academic institution were invited to participate in a pilot study to pretest the questionnaire. They did not participate in the main study. The pilot test was undertaken for three reasons: to determine whether the research instrument showed validity and reliability, whether the participants understood the questions and whether changes were required. Based on the feedback of the pilot study participants, the researchers made changes to the questionnaire. Data from the pilot study were not included in the analysis of the main study results.

### Data analysis

The data collected by means of the questionnaire were electronically transferred to a Microsoft Excel spreadsheet. The closed-ended questions were reported as descriptive statistics as percentages in tables and discussed in narrative format (Kaur, Stoltzfus & Yellapu 2018).

### Ethical considerations

The Health Sciences Research Ethics Committee (reference number: UFS-HSD2020/0367/2807) of the University of the Free State, Bloemfontein, South Africa, approved the study protocol. The researchers also obtained ethical clearance from the Free State Provincial Department of Health. In addition, in keeping with research ethics, the participants were requested to sign an informed consent form before being handed a questionnaire to complete and return to the researchers. Participation in the study was voluntary, with the option to withdraw from the study at any time. To ensure anonymity and confidentiality, a number (1–8) was allocated to each returned questionnaire.

## Results

The results of the three parts of the questionnaire are presented below.

### Part 1: Demographic information

Part 1 covered demographic variables, radiation worker status and operating forensic radiography equipment (henceforth referred to as radiography equipment) for forensic purposes. Table 3 presents the demographic data. All the participants (*n* = 8) were men. Three (37.5%) were junior registrars, one (12.5%) was a senior registrar and four (50.0%) were specialist forensic pathologists (consultants). All the participants stated that they operated the imaging machines at the selected mortuary for forensic radiography purposes. They also disclosed that they were not registered as radiation workers.

**TABLE 3:** Demographic information, qualifications and employment of participants (*n* = 8).

Variable	*n*	%
**Gender**		
Male	8	100.0
Female	0	0.0
**Age (years)**		
20–30	0	0.0
31–49	2	25.0
41–50	4	50.0
> 50	2	25.0
**Employment at the specific mortuary (years)**		
< 1	0	0.0
1–3	3	37.5
4–6	1	12.5
7–10	1	12.5
11–15	3	37.5
> 15	0	0.0
**Qualification**		
Grade 12	8	100.0
Postschool certificate	1	12.5
Postschool diploma or degree	5	62.5
Honours degree or master’s degree	4	50.0
Doctoral degree	0	0.0
**Are you registered as a radiation worker?**		
Yes	0	0.0
No	8	100.0
**Do you operate imaging equipment for forensic purposes?**		
Yes	8	100.0
No	0	0.0

There were two important reasons for using radiography equipment: to locate projectiles or foreign objects (*n* = 7; 87.5%) and to determine fractures or soft tissue injuries (*n* = 7; 87.5%). Other uses of radiography equipment were for the diagnosis of lung pathology (*n* = 1; 12.5%) and investigating the cause of death (*n* = 1; 12.5%).

A closed-ended question pertained to radiography equipment used at the mortuary. A Lodox full-body scanner was used by seven (87.5%) participants, a C-arm by two (25.0%) participants and a CT scanner by one (12.5%) participant. None of the participants indicated that they used a conventional X-ray machine, MRI scanner or conventional X-ray mobile equipment.

### Part 2: Knowledge and training of the personnel performing forensic radiography

The following issues were covered in Part 2 of the questionnaire: reasons for using radiography equipment and the type of equipment used; training on the use of radiography equipment and the type of training received; radiation protection equipment and methods of protecting self from ionising radiation; QC tests; corpse (deceased person for an autopsy) positioning training and forensic radiography image acquisition, including the use of exposure charts; use of collimation and anatomical lead markers; and reasons for the need for training, if applicable. The open-ended questions provided the opportunity for participants to list reasons as to why the imaging equipment was used, the type of training for each modality, the service providers for past training, the ways used to monitor the amount of radiation that they received and when and how QC tests were performed.

As shown in Table 4, in terms of C-arm machine training, two (25.0%) participants confirmed training. Training on the mobile digital X-ray machine was confirmed by one (12.5%) participant. Three (37.5%) participants did not indicate whether they had received training on any of the forensic radiography equipment. Two (25.0%) participants did not indicate whether they had received training on digital X-ray machines.

**TABLE 4:** Training received to operate different radiology imaging equipment.

Variable (imaging modality)	Yes		No		No indication	
	*n*	%	*n*	%	*n*	%
Conventional radiography X-ray machine	0	0.0	5	62.5	3	37.5
Digital radiography X-ray machine	0	0.0	5	62.5	3	37.5
Computed tomography	0	0.0	5	62.5	3	37.5
Magnetic resonance imaging	0	0.0	5	62.5	3	37.5
C-arm machine	2	25.0	4	50.0	2	25.0
Conventional X-ray mobile machine	0	0.0	5	62.5	3	37.5
Digital X-ray mobile machine	1	12.5	5	62.5	2	25.0

Regarding the type of training received for imaging modalities, one (12.5%) participant stated to have received workshop training on the C-arm machine. One (12.5%) participant reported about workshop training on how to use and operate a Lodox machine. In the results section, the Lodox training provided on the positioning of the corpse and the setting of exposure factors is discussed. One (12.5%) participant reported to have received training on the use of a conventional X-ray machine. The follow-up question was on the type of training received and the service provider. The training service provider was either a Lodox equipment expert or an experienced colleague at the workplace. One participant indicated ‘not applicable’ to this questionnaire item, as no training was provided on the use of any radiography imaging machine. Four (50.0%) of the participants did not answer this question.

In terms of the questions covering radiation protection training, one (12.5%) participant did not answer the question. Four (50.0%) participants indicated that they had not received radiation protection training. Three (37.5%) indicated that they had received training. Two (25.0%) received radiation protection training only for the Lodox full-body scanner; one received training in 2019 but did not indicate who provided the training, and the other participant stated the Lodox manufacturer provided training in 2020. One (12.5%) participant reported that he received training from a colleague in 1998 (more than 20 years before the study) on how to use a C-arm machine, but it is not known whether the colleague was experienced in radiation protection training. None of the three participants who had received training was issued with a training or attendance certificate for the radiation protection training.

#### Radiation protection

The participants had to select from the options provided pertaining to the type of radiation protection they used, which included full lead rubber aprons, wrap-around half-body lead shield, thyroid shield and lead glasses (Table 5). They could also indicate any other lead protection equipment not listed but they might have been using. The radiation protection most used by the participants performing forensic radiography was indicated as full lead rubber aprons (Table 5).

**TABLE 5:** Knowledge of and training on radiation protection.

Variable	*n*	%
**Concerning radiation-emitting machines (e.g. CR, CT, C-arm fluoroscopy, mobile X-ray machine), did you receive any radiation protection training for yourself as a radiation worker?**		
Yes	3	37.5
No	4	50.0
Question not answered	1	12.5
**Did you receive a training certificate or attendance certificate for the radiation protection training?**		
Yes	0	0.0
No	4	50.0
Question not answered	4	50.0
**What radiation protection equipment is used where you work?**		
Full lead apron	3	37.5
Wrap-around half-body lead shield	0	0.0
Thyroid shield	0	0.0
Lead gloves	0	0.0
Lead glasses	1	12.5
Other (specify†)	2	25.0
Question not answered	2	25.0
**Are the walls and doors of the rooms used to operate radiation-emitting machines suitable to protect those not in the room from being exposed to ionising radiation? For example, lead-lined doors or barium-coated walls, etc.**		
Yes	4	50.0
No	1	12.5
Unsure	3	37.5
**What ways are used to monitor the amount of radiation that you receive as a radiation worker?**		
Register book	1	12.5
I do not know	1	12.5
Wearable tag, dosimeter or radiation exposure monitor	4	50.0
Register of time spent using the Lodox machine	2	25.0
None	1	12.5

CT, computed tomography.

† , Lead-lined cubicle or shield wall.

In terms of monitoring the amount of ionising radiation that they received when performing forensic radiography, four (50.0%) indicated that they used a wearable tag, dosimeter or radiation exposure monitor (see Table 5). A register book was also used to record the amount of radiation exposure. One (12.5%) did not know how monitoring the amount of radiation exposure was achieved, while another one (12.5%) indicated there was no available method used to monitor radiation exposure at the forensic mortuary.

#### Quality control on forensic equipment

As shown in Table 6, only one (12.5%) participant indicated that QC tests were performed for each piece of X-ray equipment available at the research site. Three (37.5%) participants stated that QC tests were not done, and four (50.0%) were not sure about this. The participants performing forensic radiography were asked to specify what type of QC tests were performed on the imaging equipment. One (12.5%) participant indicated that ‘regular inspection and service by Lodox’ was considered a QC test method. The remainder (*n* = 7; 87.5%) were not sure (*n* = 1; 12.5%), did not answer the question (*n* = 4; 50%) or indicated not applicable (*n* = 2; 25%). By implication, they either did not know or were unsure whether QC tests were performed. One (12.5%) participant indicated that the service provider was responsible for performing the QC tests of the equipment. In terms of the frequency of QC tests being performed, two (25.0%) participants were unsure, and one did not answer the question.

**TABLE 6:** Responses related to knowledge of and training on quality control.

Question	*n*	%
**Are there any quality control tests performed on the machine you use?**		
Yes	1	12.5
No	3	37.5
Unsure	4	50.0
**Name the quality control tests that are performed for each of the X-ray equipment you have available.**		
Not applicable	2	25.0
Regular inspection and service by Lodox	1	12.5
Not sure	1	12.5
Did not answer the question	4	40.0
**Who is responsible for performing the quality control (QC) tests of the equipment?**		
Not applicable	1	12.5
Service provider	1	12.5
I do not know	1	12.5
Did not answer the question	5	62.5
**When are the QC tests performed (frequency – daily, weekly, monthly)?**		
Not applicable	1	12.5
Unsure – quarterly	1	12.5
Not sure	1	12.5
Did not answer the question	5	62.5
**Did you receive training regarding the QC monitoring of an X-ray machine?**		
Yes	0	0.0
No	7	87.5
Did not answer the question	1	12.5
**If yes, please provide more information indicating the type of training and the service provider.**		
Type of training: did not answer the question	8	100
Service provider: did not answer the question	8	100

#### Positioning and exposure selection

The results regarding the positioning of cadavers for image acquisition showed that the majority (*n* = 7; 87.5%) had received training on positioning, and all stated that the Lodox equipment expert provided the training. In terms of training in the setting of exposure factors, namely kVp and mAs or automatic exposure settings, the majority (*n* = 6; 75.0%) had not received training. Two (25.0%) participants said they had received training from an equipment expert at a Lodox workshop. Four (50.0%) confirmed that they had exposure charts that they used as guidance when setting exposures. Two (25.0%) were not sure, and one (12.5%) stated that no exposure charts were available to refer to. Regarding the updating of exposure charts, four (50.0%) answered the question: one (12.5%) was not sure, one (12.5%) stated the chart was updated in 2019, one (12.5%) stated the chart was usually updated when the machine was serviced and one (12.5%) stated that it was ‘part of the computer software’ of the Lodox full-body scanner.

The majority (*n* = 7; 87.5%) of participants answered the question regarding departmental imaging protocols for performing forensic radiography: two (25%) confirmed there were protocols, and two (25%) stated there were none. The remaining three (37.5%) were not sure about the use of protocols. Follow-up options to select from were available for the two participants who confirmed that there were protocols: exposure factors, positioning criteria that should be followed and which anatomical areas should be included. They selected anatomical areas that should be included (25.0%), that positioning criteria must be followed (25.0%) and one (12.5%) selected exposure factors.

#### Lead marker placement

Five (62.5%) participants answered the question whether they used anatomical lead marker placement. Two (40.0%) of these participants confirmed that they did, while the others indicated that did not use lead markers.

#### Areas in which forensic radiography training is needed

The participants were asked to provide suggestions with reasons regarding areas in which they would like to receive training. Three (37.5%) participants wanted training on radiation protection because they had not received adequate training, including on how to safely use the machine. Two (25.0%) participants indicated that they would like training on positioning of the corpse for image acquisition to obtain the best possible quality forensic radiography images. Five (62.5%) participants stated the need for training on different forensic radiography protocols because they had not received such training. Four (50.0%) participants expressed an interest in receiving training on how to conduct QC tests on the forensic radiography equipment they used at the forensic mortuary. They provided two reasons for receiving training on QC: they had not received training before, and they wanted to know how to perform these tests to help extend the life of the radiography equipment. Four (50.0%) participants indicated a need for training regarding forensic radiography exposure factor settings because they had not received adequate training. Two (25.0%) participants wanted training on X-ray machine operation. One (12.5%) participant indicated the need for training on all aspects to ensure using the machine safely, to obtain the best possible quality forensic radiography images and to extend the operational life of the Lodox for as long as possible. Table 7 provides a summary of the areas in which a need for training was identified for the personnel performing forensic radiography at the selected study site.

**TABLE 7:** Areas in which training is needed (*n* = 8).

Training area	*n*	%
Radiation protection	3	37.5
Corpse positioning	2	25.0
Different forensic imaging protocols	5	62.5
Quality control of imaging equipment	4	50.0
Exposure factors	4	50.0
Machine operation	2	25.0
Other	1	12.5

### Part 3: Comments and feedback

The last question was open-ended to allow the participants to provide further comments or feedback. Comments were received from Participants 5 and 6. Participant 5 stated, ‘[*i*]t looks to me that the design of the machine [*Lodox*] was so designed so simple as to be used by untrained people’. Participant 6 stated, ‘[*t*]he company providing the imaging equipment should provide thorough training’.

## Discussion

The aim of this study was to evaluate the knowledge and training of personnel performing forensic radiography at a mortuary. Radiographers are skilled in both obtaining X-ray images and applying information that would assist to control the patient (Tarani et al. 2017). As discussed above, this specific accessible population did not include radiographers. According to the Department of Health, a radiation worker is anyone who works in an occupation that brings them into contact with more than three-tenths of the occupational dose limit (Department of Health 2014). A pathologist, therefore, is a radiation worker who may operate X-ray equipment for nonmedical purposes (Department of Health 2014).

The identified radiation worker is then issued with personal radiation monitoring devices (PRMDs). A PRMD continuously monitors the environment and the dosage of radiation that their bodies have been exposed to. Contrary to this information, all the participants (*n* = 8) indicated that they were not registered radiation workers, and four (50.0%) of them used radiation monitoring devices. Personal radiation monitoring devices are issued to radiation workers in South Africa by the South African Bureau of Standards (SABS) Holdings (Pty) Ltd. Radiation Protection Services, and each monitoring device has a unique Bureau Identification Number (BIN) that is allocated by Radiation Protection Services to a specific registered radiation worker. The PRMD must be replaced in less than 32 days, and records of the exposure must be kept on record by the service provider for 10 years (Department of Health 2014). Hence, it might be possible that the participants were not adequately informed that they were considered to be radiation workers.

In a study performed on the experiences of forensic pathologists and radiographers in providing forensic radiography services, the findings indicated that the participants did not receive any training on forensic radiography. Furthermore, they also indicated that there was no forensic radiography available and that they had to learn on the job (Sangonuga et al. 2022). In terms of the current study performed in the Free State province (South Africa), only three (37.5%) participants stated that they had received training to operate forensic radiography equipment. One (12.5%) of the participants indicated that training was received to operate the C-arm machine. This finding emphasises the need for training to adhere to the requirement that the licence holder of X-ray machine equipment must ensure that all personnel using the equipment must be trained (Department of Health 2014).

Six (75.0%) participants had not received training on technical factors, such as exposure settings. The majority (*n* = 7; 87.5%) of participants stated that the Lodox full-body scanner was the most frequently used machine, followed by the C-arm machine (*n* = 2; 25.0%). Therefore, exposure setting would not be required as these two machines employ automatic exposure control. Because of the COVID-19 pandemic, the planned Lodox training at this site was postponed.

Radiation workers are provided with appropriate protective clothing and safety instructions and guidelines (Department of Health 2016). All forensic personnel within 1 m of an X-ray tube or a corpse must wear protective clothing with the specific lead equivalence of 0.35 mm Pb plus a thyroid shield and leaded glasses (Department of Health 2014). It is important to highlight that not all participants in this study were aware that they should use protective gear when using radiation-emitting devices.

Half of the participants expressed an interest in receiving training on how to conduct QC tests on the forensic radiography equipment they used at the forensic mortuary. The use of X-ray equipment is controlled by the *Hazardous Substances Act* 15 of 1973 (DoH 1973, DoH 2016). This legislation requires that an inspection body, approved by the Department of Health, must perform an initial acceptance test of all X-ray equipment and regularly perform safety testing (DoH 2016). Every machine must be licensed, and the licence holder of X-ray equipment must appoint in writing an appropriately trained person to take responsibility for the safe use of the equipment, training of staff members and that QC tests are performed (DoH 2016). The equipment used for nonmedical forensic purposes must also be maintained by the licence holder and responsible person and the safe use of the equipment must be monitored on a regular basis. A register must be kept of the X-ray examinations with specific reference to the name of the operator and the deceased, the date, the reason for the examination and the dose or exposure time of the procedure. Quality control of the equipment is mentioned as record-keeping for inspection pertaining to the service record of the equipment and the protective clothing that must be tested twice a year. The code further indicates that records must be available for inspection by the DRC (Department of Health 2014). The researchers therefore assume that records of training and QC tests will be maintained by the licence holder and responsible person for inspection by the DRC.

About one-fourth (*n* = 2; 25.0%) of the participants indicated that protocols were not followed when performing forensic radiography. Three (37.5%) participants were unsure or unaware of forensic radiography imaging protocols. It could be argued that they either did not know about forensic radiography imaging protocols or were unaware because they had not received any formal training. Aalders et al. (2017) emphasised that the forensic radiography protocol applied for acquiring images affects image quality. Furthermore, Flach et al. (2014) emphasised that personnel performing forensic radiography on advanced imaging equipment, such as CT, should adhere to medicolegal requirements in terms of image quality, protocols and standards. Furthermore, the importance of best practice in forensic imaging, including medicolegal requirements, has been emphasised by Doyle et al. (2020).

The participants at the selected site performed forensic radiography to determine the cause of death or for judiciary purposes. For authentication purposes, all radiography images must have a correct anatomical lead (side) marker according to best practice standards (Johnson 2014; Sebelego, Van der Merwe & Du Plessis 2019). The date of imaging should be clearly recorded so that the person who acquired the image might be held accountable in both criminal and civil cases, if necessary (Johnson 2014). It is evident that the use of such markers needs to be highlighted in the areas in which training is required. Three (37.5%) of the participants were unaware that the placement of lead markers was a part of forensic records. As three (37.5%) participants did not answer this question, it would be reasonable to assume that they were either unsure about or did not know this.

## Limitations of the study

The main limitation of this study was its small sample size. Currently, no records about the total number of personnel performing forensic radiography in South African forensic pathology mortuaries are available. The use of purposive nonprobability sampling is a limitation in the research. The results should not be regarded as representative of the target population, which, in this study, was medical personnel, excluding radiographers, who performed forensic radiography for forensic purposes. Additionally, statistical analysis was limited because of the sample size. The lack of response to several questions was also a limitation. Limited personal contact occurred between the researchers and the participants during the data collection because of COVID-19 pandemic regulations, and the envelope containing the completed questionnaires was collected from the receptionists. Test–retest reliability could not be performed by distributing the questionnaire at two different time points because of the high workload of the participants (Morrison n.d.).

## Recommendations

The unavailability of radiographers at a forensic pathology mortuary for forensic radiography is a reality, but the authors strongly recommend that radiographers should be engaged on a monthly basis to at least assist the mortuary with the routine QC tests. Radiographers could be responsible for the training of the forensic operators to ensure the accurate use of equipment, protective clothing and record-keeping, as inspections by the DRC occur at longer intervals. Providers of medical equipment should be responsible for ensuring that the training is documented so that a proof is available for the inspectors of the DRC. The ethical principles of beneficence, nonmaleficence and justice should be adhered to in forensic radiography. In other words, it is essential that personnel who perform forensic radiography in forensic pathology mortuaries must ensure all aspects of best practice at all times.

It is further recommended that an online programme or a short course should be developed to address the required training needs outlined in this study. The researchers recommend that a multicentre comparative study should be conducted at other state forensic facilities in South Africa to determine whether there is a general need for training of personnel performing forensic radiography in forensic pathology mortuaries.

## Conclusion

The findings of this study show that the participants lacked knowledge and training regarding forensic radiography at the selected research site. They indicated a need for training on radiation protection, corpse positioning for image acquisition and different forensic radiography imaging protocols. They also expressed the need for training in QC tests that should be performed on the radiography equipment. They indicated that performing QC tests should contribute to extending the life of the expensive radiography equipment they use. They also need training in protecting themselves and the public against ionising radiation. They need training to ensure that imaging equipment is used to its full intended capacity. Although performed on a small scale, this study sets the scene for future research including more participants to determine the knowledge and training needed for personnel performing forensic radiography in South Africa. The results of this study could be used by others in the health departments in South Africa to identify areas of training required to provide forensic radiography services.
